# Oral Rehabilitation with Zygomatic Implants in a Patient with Cleft Palate

**DOI:** 10.1155/2019/6591256

**Published:** 2019-05-06

**Authors:** Guilherme José Pimentel Lopes de Oliveira, Mariana Schaffer Brackmann, Larissa Carvalho Trojan, Paulo Domingos Ribeiro Júnior, Luis Eduardo Marques Padovan

**Affiliations:** ^1^Department of Periodontology/Implantology, Dental School, Federal University of Uberlândia-UFU, Uberlândia 38405-266, Brazil; ^2^Department of Diagnosis and Surgery, Univ. Est. Paul.-UNESP, Araraquara 14801-903, Brazil; ^3^Instituto Latino Americano de Ensino e Pesquisa Odontológico-ILAPEO, Curitiba 80710-150, Brazil; ^4^Univ. Sagrado Coração, Bauru 17011-160, Brazil

## Abstract

Edentulous patients with an atrophic maxilla associated with lip-palate fissures have unpredictable results after undergoing grafting procedures. In situations where the atrophic maxilla does not adequately allow reconstruction, the use of zygomatic implants has been indicated, and probably these implants can be indicated for the rehabilitation of patients with lip-palate fissures. This case report describes the oral rehabilitation treatment of a patient with a lip-palate cleft treated with zygomatic implants and implant-supported fixed prosthesis with two years of follow-up. A 65-year-old female patient had a lip-palate cleft and previously underwent surgery to close the cleft. The patient had a severely atrophic maxilla and had difficulty adapting to a removable total prosthesis. Due to the small amount of bone remaining and extensive fibrous tissue in the palate region, a rehabilitation with conventional implants associated with zygomatic implants was chosen. Two zygomatic implants and a conventional implant were placed on the right side, and a zygomatic implant and conventional implant were placed on the left side; these implants were later activated by a protocol-type prosthesis. The zygomatic implants provided an adequate aesthetic and functional outcome of the prosthesis in a patient with cleft palate.

## 1. Introduction

The use of implant-supported prostheses has been shown to be a predictable treatment with a high success rate for the treatment of any type of edentulism [1, 2]. However, in some clinical situations, it is not possible to place implants in a good position due to the limited availability of the bone tissue [3, 4]. In these situations, the rehabilitation of the atrophic maxilla may represent the greatest challenge because it is a bone with low biological quality compared to the mandible and contains a sinus cavity that tends to increase its degree of pneumatization after tooth loss [3, 5].

To address this problem, bone substitute biomaterials associated with maxillary sinus floor elevation techniques [6], guided bone regeneration [5], osteogenic distraction [4], and the use of block grafts [7] have previously been proposed to treat the atrophic maxilla before implant placement. However, in some cases, maxillary atrophy may be of a high degree of severity that renders bone reconstruction procedures unpredictable.

Edentulous patients with an atrophic maxilla associated with lip-palate fissures have unpredictable results after undergoing grafting because they have a compromised local vascularization, low tissue elasticity, and impaired psychological aspects. In addition, the limitation of the openness of the mouth makes it difficult to place implants correctly [8]. In situations where the atrophic maxilla does not adequately allow reconstruction, the use of zygomatic implants has been indicated. These implants were originally designed for the treatment of maxillectomized patients who were tumour or trauma victims [9, 10] and may be an excellent alternative in the rehabilitative treatment of patients with lip-palate fissures [11].

This case report is aimed at describing and presenting the outcomes of the oral rehabilitation treatment of a patient with a lip-palate cleft who was treated with zygomatic implants and implant-supported fixed prosthesis after two years of follow-up.

## 2. Case Presentation

**Figure 1:**
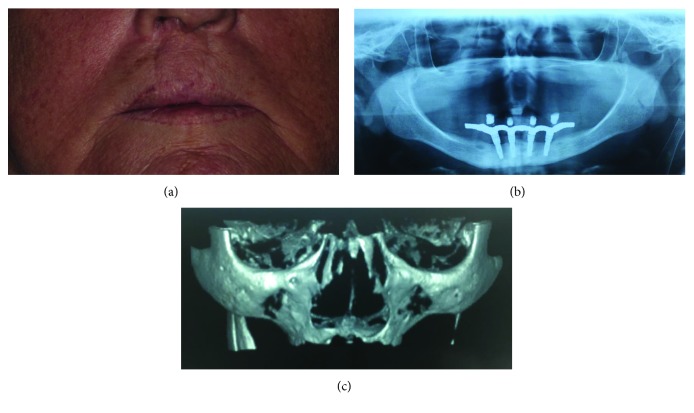
(a) Preoperative clinical condition of the patient, (b) preoperative panoramic radiography, and (c) preoperative cone beam computed tomography. Note the large degree of maxilla atrophy presented by the patient before the surgical procedure.

A 65-year-old female patient who presented with a lip-palate cleft previously underwent a surgical procedure to close the lip-palate cleft. However, even after this procedure, the patient had a clearly atrophic maxilla (class V of Cawood and Howell) and had difficulty adapting to a total removable of prosthesis. Due to the small amount of bone remaining and extensive fibrous tissue in the palate region, rehabilitation with conventional implants associated with zygomatic implants was chosen instead of subjecting the patient to a reconstruction with large bone grafts (Figure 1(a)). For the preoperative evaluation, panoramic radiography and cone-beam computed tomography of the maxilla and zygomas were requested, which confirmed the low bone availability in this case (Figures 1(b) and 1(c)). Two zygomatic implants and a conventional implant were placed in the right side, and one zygomatic implant and one conventional implant were placed in the left side under general anaesthesia and nasotracheal intubation. Zygomatic implants with the Cone Morse platform (Neodent®, Curitiba, Brazil) and conventional implants with the Cone Morse platform and a hydrophilic surface (Acqua surface, Neodent®, Curitiba, Brazil) were used. Of these implants, a zygomatic implant with 4.4 × 52.5 mm was installed in the region of tooth 12, a 4.4 × 40 mm zygomatic implant was installed in the region of tooth 16, a zygomatic implant with 4.4 × 52.5 mm was installed in the region of tooth 26, and two conventional conical implants with 3.5 × 11.5 mm were placed in the region of teeth 14 and 23. Furthermore, more than 60 N·cm of insertion torque was obtained during the placement of all the implants.

**Figure 2:**
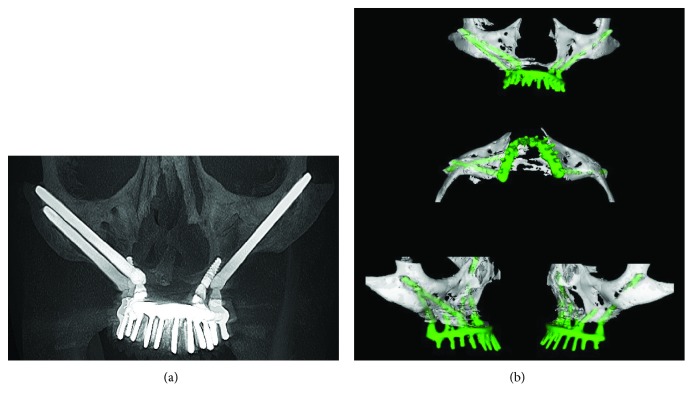
(a) Panoramic tomography and (b) tomographic image of the patient after 2 years of follow-up. Note the good position of the implants placed and the absence of complications.

**Figure 3:**
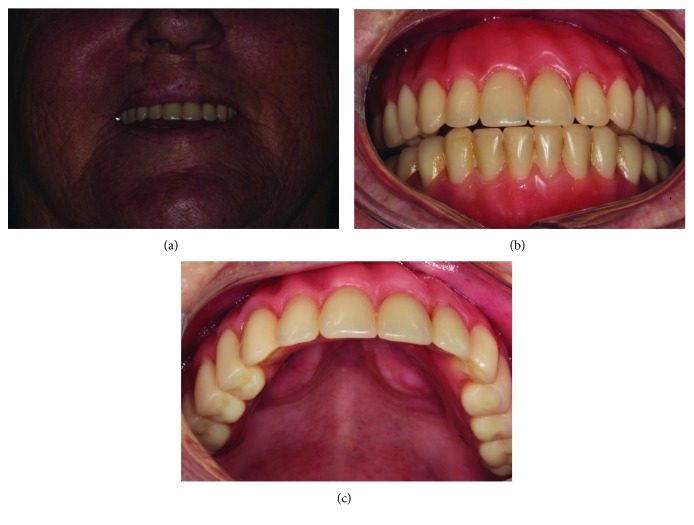
(a) Postoperative clinical condition of the patient with the upper protocol-type prosthesis in function, (b) postoperative condition of the prosthesis, and (c) postoperative condition of the palate. Note the good clinical outcome of the upper protocol-type prosthesis after 2 years of follow-up. Furthermore, a good condition of the palate with the communication of the nasal and oral cavities is showed.

During the milling of the surgical site, the following sequence of drills was used: spherical drill zygomatic plus 2, spiral drill 2.7 zygomatic plus, pilot spiral drill zygomatic plus 2.7/3.3, spiral drill zygomatic plus 3.3, pilot spiral drill zygomatic plus 3.3/3.7, and countersink drill for Cone Morse zygomatic implant placement, and then the implant installation was performed. A surgical guide was used during the implant surgical site preparation. An antibiotic (amoxicillin) was given intraoperatively and maintained for seven days. Miniabutments were selected and installed at the time of surgery. After 24 hours, the castings were performed, and 48 hours after surgery, a screwed fixed-implant protocol prosthesis with CrCo infrastructure and acrylic teeth was installed on the implants. During the postoperative period, radiographic and clinical examinations showed that there were no complications (Figures 2(a) and 2(b)). The patient underwent follow-up after 15, 30, and 90 days and then every six months thereafter. Currently, the patient has been followed up for two years without any complaint and with a functional prosthesis (Figures 3(a)–3(c)). Furthermore, the patient expressed satisfaction with the obtained aesthetic and functional outcome. The patient gave the researchers a signed consent for the publication of this case.

## 3. Discussion

In general, it was verified in this case report that the use of zygomatic implants was effective for providing a basis for the rehabilitative treatment of a patient who presented with a lip-palate cleft. As the patient had already undergone grafting procedures and because she was not of ideal age to undergo other grafting procedures to reduce the fibrous tissue present on the palate, the grafting technique was contraindicated for the use of zygomatic implants.

Lip-palate clefts are the most frequent craniofacial malformations. These deformities were considered by the World Health Organization to be a public health problem. In 2002, this type of malformation accounted for 1 in 650 babies born worldwide [12]. The fissures present a multifactorial aetiology, with the involvement of genetic and environmental factors. Treatment of patients affected by these malformations should begin soon after birth and extend into adulthood [13]. As the patient of the present case report did not undergo treatment in the early stage, there was a reduced chance of success with the grafting procedures.

Zygomatic implants were initially designed to be used to treat atrophic maxilla to avoid graft procedures; these implants reduce the degree of morbidity of the surgical procedure and costs and accelerate prosthetic rehabilitative treatment [14]. However, it has been reported that the success of installing zygomatic implants is related to the degree of experience and skill of the surgeon and that some complications, such as haemorrhages during the procedure or the occurrence of sinusitis after the procedure, are not uncommon [15]. On the other hand, some studies have shown that zygomatic implants have high success and survival rates [10, 14, 16], which shows that rehabilitation treatment with this type of implant is safe, as confirmed in the case report described. In this case report, the indication of the use of the zygomatic implant was due to the lacking of enough bone tissue which enables the placement of an adequate number of implants of conventional size suitable for the installation of a fixed-implant protocol prosthesis due to atrophy of the maxilla associated with the presence of the cleft palate.

It has been a tendency in medical areas to indicate less invasive surgical procedures that are associated with lower morbidity for patients [17, 18]. Less invasive procedures accelerate the healing process and generate a high degree of satisfaction of patients, and whenever possible, less invasive surgical procedures should be indicated. Although grafting procedures are related to high rates of clinical success, this additional surgical step requires more time due to implant placement in most cases [14, 16]. In addition, in cases of severe atrophy, the use of an autogenous bone graft isolated or associated with other bone substitutes is more appropriate because it is necessary that the graft presents good biological properties to integrate into the surgical beds of the native bone with little thickness [5]. In these cases, morbidity in the donor sites generates a great discomfort to patients [19]. In fact, the option to install zygomatic implants in this case promoted the application of a more conservative surgical technique with a smaller possibility of causing morbidity to the patient and promoted acceleration of prosthetic rehabilitation, thus promoting a greater patient satisfaction with the surgical procedure performed.

Another possibility for rehabilitation of this case would be the use of overdenture-type prosthesis that has been shown to be a conservative treatment with high rates of success and patient satisfaction [20]. However, in this specific case, the patient's previous positive experience with the protocol-fixed prostheses used to rehabilitate the inferior jaw was taken into account. In addition, there was no communication of the nasal with the buccal cavity due to the grafting attempt that sealed this communication.

## 4. Conclusion

This case report showed that the use of zygomatic implants in a severely atrophic maxilla provides a sufficiently stable support for the installation of a total-fixed prosthesis in the atrophic maxilla of a lip-palate cleft patient with good clinical outcomes after two years of follow-up.
